# Near-Complete Genome Assembly of the Grapevine Crown Gall Pathogen Allorhizobium vitis Strain K377

**DOI:** 10.1128/MRA.01359-20

**Published:** 2021-09-30

**Authors:** Hangwei Xi, Maarten Ryder, Iain R. Searle

**Affiliations:** a School of Biological Sciences, The University of Adelaide, Adelaide, Australia; b School of Agriculture, Food and Wine, The University of Adelaide, Adelaide, Australia; University of California, Riverside

## Abstract

Here, we report the annotated, near-complete genome sequence of Allorhizobium vitis K377, a phytopathogenic *Rhizobiales* strain isolated from a grapevine in South Australia. The assembled genome sequence is 6.40 Mb long, with 5,855 predicted protein-coding sequences, 56 tRNAs, and 12 rRNAs, and contains *ttuC* (tartrate metabolism; chromosomal) and nopaline synthesis, uptake, and catabolic genes (tumor-inducing plasmid-encoded).

## ANNOUNCEMENT

Grapevine crown gall is a serious chronic disease that can cause decreased production or grapevine death (1, 2). The causal agent of crown gall on grapevine is Agrobacterium vitis, now reclassified into the genus *Allorhizobium* (3). Virulent Allorhizobium vitis strains harbor a tumor-inducing plasmid (pTi) that upon infection integrates the transferred DNA (T-DNA) region into the plant genome under the mediation of the vir (virulence) proteins (4). The T-DNA genes mainly encode two kinds of products: (i) auxins, which promote cell division and result in gall formation (5), and (ii) opines, which can be metabolized by some *A. vitis* strains and used as an energy source (6). To date, the complete genome sequences of *A. vitis* S4 (7) and K306 (8) are the only 2 reported complete *A. vitis* genome sequences.

*A. vitis* K377 was first isolated from a cv. Ramsey grapevine at Nuriootpa, South Australia, in 1979 (9). A single colony of K377 (Kerr collection, University of Adelaide) was inoculated into 5 ml yeast mannitol broth and grown overnight at 28°C. Genomic DNA purification was performed using a PowerSoil kit (Qiagen). After shearing the purified DNA to 10 to 15 Kb with a 26-gauge needle, the Blue Pippin system (Sage Science) was used to select fragments between 10 and 25 Kb. For PacBio library preparation and sequencing, DNA was processed using the SMRTbell template prep kit v1.0 (PacBio, Menlo Park, CA) and sequenced on a PacBio Sequel instrument. A total of 1.65 Gb sequencing data with a read *N*_50_ of 6.79 Kb was obtained, representing approximately 250× coverage.

A genome assembly was built from the PacBio sequence reads using Flye v2.8.1 (10) in metagenome mode. The assembly consisted initially of seven contigs with an *N*_50_ value of 3.79 Mb. The PacBio reads were mapped back to the draft genome assembly using Minimap2 v2.1 (11), and the mapping rate was 98.6%. BUSCO v4.1.3 (12) identified 96.7% complete genes in the assembly out of 1,937 markers in the rhizobium-agrobacterium_group_odb10 database, suggesting high integrity and completeness of the assembly. Three of the seven contigs were demonstrated as circular sequences by manually linking the 3′ and 5′ ends and identifying spanning sequence reads using Minimap2 (Geneious Prime v2020.2.4). LASTZ (13) was used to identify the similarity of each contig with closely related bacterial species using default parameters (step length = 20, seed pattern = 12 of 19, HSP threshold score = 3000; Geneious Prime plugin LASTZ v1.02.00). Three contigs were very similar (average nucleotide identity [ANI], >95%) to *A. vitis* strain S4 chromosome 1, chromosome 2, and plasmid pAtS4e (GenBank accession number GCA_000016285.1). Two other contigs were partially similar (ANI, >85%) to the C58 plasmid Ti (NC_003065.2) using FastANI v1.32. Genome annotation was performed using the DDBJ Fast Annotation and Submission Tool (DFAST) (14).

The final, high-quality draft genome sequence of seven contigs has a total length of 6.40 Mb, an *N*_50_ value of 3.79 Mb, and a GC content of 57.6%. DFAST identified 5,855 protein-coding sequences, 56 tRNAs, and 12 rRNAs (Table 1). The *virC* virulence gene and T-DNA endonuclease VirD1 were identified on contig 2, indicating that contig 2 is likely to be a part of the tumor-inducing (Ti) plasmid pTiK377. The complement and alignment of T-DNA genes in pTiK377 is indistinguishable from that of other *A. vitis* Ti plasmids of type IVa (15). Genes related to nopaline uptake and metabolism were identified on contigs 1, 2, and 4, strongly suggesting that strain K377 is nopaline metabolizing, and this is the first high-quality genomic resource for strains of *A. vitis* that metabolize nopaline.

**TABLE 1:** Genomic features of *A. vitis* K377

Characteristic	Contig 1	Contig 2	Contig 3	Contig 4	Contig 5	Contig 6	Contig 7
Function (plasmid)	Nopaline uptake and metabolism (pAvK377)	Tumor induction, nopaline and agrocinopine uptake and metabolism (part of pTiK377)	Chr 2^*a*^ (ttuC)	Nopaline uptake	Chr 1^*a*^		
Size (bp)	187,327	152,864	1,593,087	620,734	3,787,628	56,347	3,065
Circular	Yes	No	Yes	No	Yes	No	No
GC content (%)	59.0	56.3	57.5	56.7	57.8	57.3	57.3
No. of CDS^*b*^	181	146	1,405	564	3,495	59	5
No. of rRNAs	0	0	3	0	9	0	0
No. of tRNAs	0	0	4	0	52	0	0

a Chr, chromosome.

b CDS, coding DNA sequences.

### Data availability.

The complete genome sequence and associated data for *A. vitis* K377 were deposited under GenBank accession number JACXXJ000000000, BioProject accession number PRJNA664275, SRA accession number SRR12701095, and BioSample accession number SAMN16204844.
